# Pregnancy outcomes in women with gestational diabetes mellitus diagnosed according to the WHO-2013 and WHO-1999 diagnostic criteria: a multicentre retrospective cohort study

**DOI:** 10.1186/s12884-018-1810-5

**Published:** 2018-05-10

**Authors:** Eva A. R. Goedegebure, Sarah H. Koning, Klaas Hoogenberg, Fleurisca J. Korteweg, Helen L. Lutgers, Mattheus J. M. Diekman, Eva Stekkinger, Paul P. van den Berg, Joost J. Zwart

**Affiliations:** 1 Department of Obstetrics and Gynaecology, Deventer Hospital, Deventer, the Netherlands; 2 Department of Endocrinology, University of Groningen, University Medical Center Groningen, PO Box 30.001, 9700 RB Groningen, the Netherlands; 3 Department of Internal Medicine, Martini Hospital, Groningen, the Netherlands; 4 Department of Obstetrics and Gynaecology, Martini Hospital, Groningen, the Netherlands; 5 Department of Internal Medicine, Medical Center Leeuwarden, Leeuwarden, the Netherlands; 6 Department of Internal Medicine, Deventer Hospital, Deventer, the Netherlands; 7 Department of Obstetrics and Gynaecology, University of Groningen, University Medical Center Groningen, Groningen, the Netherlands

**Keywords:** Gestational diabetes mellitus, GDM, WHO, Diagnostic criteria, Pregnancy outcomes

## Abstract

**Background:**

The World Health Organization (WHO) adopted more stringent diagnostic criteria for GDM in 2013, to improve pregnancy outcomes. However, there is no global consensus on these new diagnostic criteria, because of limited evidence. The objective of the study was to evaluate maternal characteristics and pregnancy outcomes in two cohorts in the Netherlands applying different diagnostic criteria for GDM i.e. WHO-2013 and WHO-1999.

**Methods:**

A multicenter retrospective study involving singleton GDM pregnancies in two regions, between 2011 and 2016. Women were diagnosed according to the WHO-2013 criteria in the Deventer region (WHO-2013-cohort) and according to the WHO-1999 criteria in the Groningen region (WHO-1999-cohort). After GDM diagnosis, all women were treated equally based on the national guideline. Maternal characteristics and pregnancy outcomes were compared between the two groups.

**Results:**

In total 1386 women with GDM were included in the study. Women in the WHO-2013-cohort were older and had a higher pre-gestational body mass index. They were diagnosed earlier (24.9 [IQR 23.3–29.0] versus 27.7 [IQR 25.9–30.7] weeks, *p* = < 0.001) and less women were treated with additional insulin therapy (15.6% versus 43.4%, *p* = < 0.001). Rate of spontaneous delivery was higher in the WHO-2013-cohort (73.1% versus 67.4%, *p* = 0.032). The percentage large-for-gestational-age (LGA) neonates (birth weight > 90th percentile, corrected for sex, ethnicity, parity, and gestational age) was lower in the WHO-2013- cohort, but not statistical significant (16.5% versus 18.5%, *p* = 0.379). There were no differences between the cohorts regarding stillbirth, birth trauma, low Apgar score, and preeclampsia.

**Conclusions:**

Using the new WHO-2013 criteria resulted in an earlier GDM diagnosis, less women needed insulin treatment and more spontaneous deliveries occurred when compared to the cohort diagnosed with WHO-1999 criteria. No differences were found in adverse pregnancy outcomes.

## Background

Gestational diabetes mellitus (GDM) is defined as glucose intolerance detected during pregnancy [1]. The prevalence of GDM is increasing and affects between 1 and 14% of all pregnancies, caused by a global increase in the number of women with obesity around reproductive age and by more stringent diagnostic criteria for GDM [1–4]. Untreated GDM is associated with an increased rate of neonatal and obstetric complications [5–7]. Adverse pregnancy outcomes have been shown to improve with timely diagnosis and treatment of GDM [8].

In 2008, the international prospective Hyperglycemia and Adverse Pregnancy Outcomes (HAPO) study group demonstrated a continuous association between maternal hyperglycaemia and risk of adverse pregnancy outcomes, as birth weight greater than the 90th percentile, caesarean section, premature birth, birth injury, and preeclampsia [9]. Based on these findings and earlier observational studies, the International Association of Diabetes and Pregnancy Study Group (IADPSG) proposed more stringent diagnostic thresholds for GDM [10]. These new diagnostic criteria (fasting plasma glucose level ≥ 5.1 mmol/l and/or 1-h plasma glucose level ≥ 10.0 mmol/l and/or 2-h plasma glucose level ≥ 8.5 mmol/l) have been adopted by the American Diabetes Association in 2010, the World Health Organization (WHO) in 2013, and the International Federation of Gynaecology and Obstetrics in 2015 [1, 11, 12].

However, to date there is no global consensus on these new diagnostic criteria. A recent review on the current European situation showed a lack of consistency on GDM diagnosis [13]. The apparent reluctance to adopt the IADPSG criteria may result from studies showing an increase in prevalence of GDM and thus a higher burden to obstetric healthcare providers [4, 14], but most importantly from scepticism about the clinical benefit of lower diagnostic thresholds [14, 15].

Also in the Netherlands there is a debate regarding the diagnostic criteria for GDM. The Dutch Society of Obstetrics and Gynaecology guideline 2010 “Diabetes and Pregnancy” recommends screening for GDM in high-risk women using the 2-h 75-g oral glucose tolerance test (OGTT) using the older WHO-1999 criteria, utilizing a fasting blood glucose ≥7.0 and 2-h blood glucose of ≥7.8 mmol/l [16, 17]. Notwithstanding that, a few hospitals in the Netherlands already implemented the new WHO-2013 thresholds for diagnosis of GDM.

To verify the consequences of implementing these new WHO-2013 thresholds the following question need to be answered: What are the pregnancy outcomes of women diagnosed according the WHO-2013 criteria compared with women diagnosed according the older WHO-1999 criteria?

The objective of the current study was therefore to evaluate the maternal characteristics and obstetric and neonatal outcome in two typical population-based cohorts in the Netherlands which applied the two different diagnostic criteria for GDM i.e. WHO-2013 and WHO-1999.

## Methods

### Study population

A multicentre, retrospective cohort study was conducted involving three hospitals in the Netherlands (University Medical Center Groningen a tertiary care centre, Martini Hospital Groningen, and Deventer Hospital both secondary care centres). Both regions (Deventer region and Groningen region) are located in the relatively rural north-eastern part of the Netherlands. Part of the data of the Groningen region has been published previously [18, 19]. All pregnant women with diagnosis of GDM were eligible for inclusion in the study. Women with a twin pregnancy and women with pre-existing diabetes mellitus (DM) were excluded.

This study has been conducted in accordance with the guidelines of the Declaration of Helsinki and Good Clinical Practice. The patient data were retrospectively acquired from hospital records generated during care-as-usual. Statistical analysis was performed requiring patient anonymity in agreement with the ethics committee regulations [20]. According to the Dutch law on Medical Research with Human Subjects, this study has been exempted for approval by the local ethics committees.

### Screening, diagnosis and treatment of GDM

**Fig. 1:**
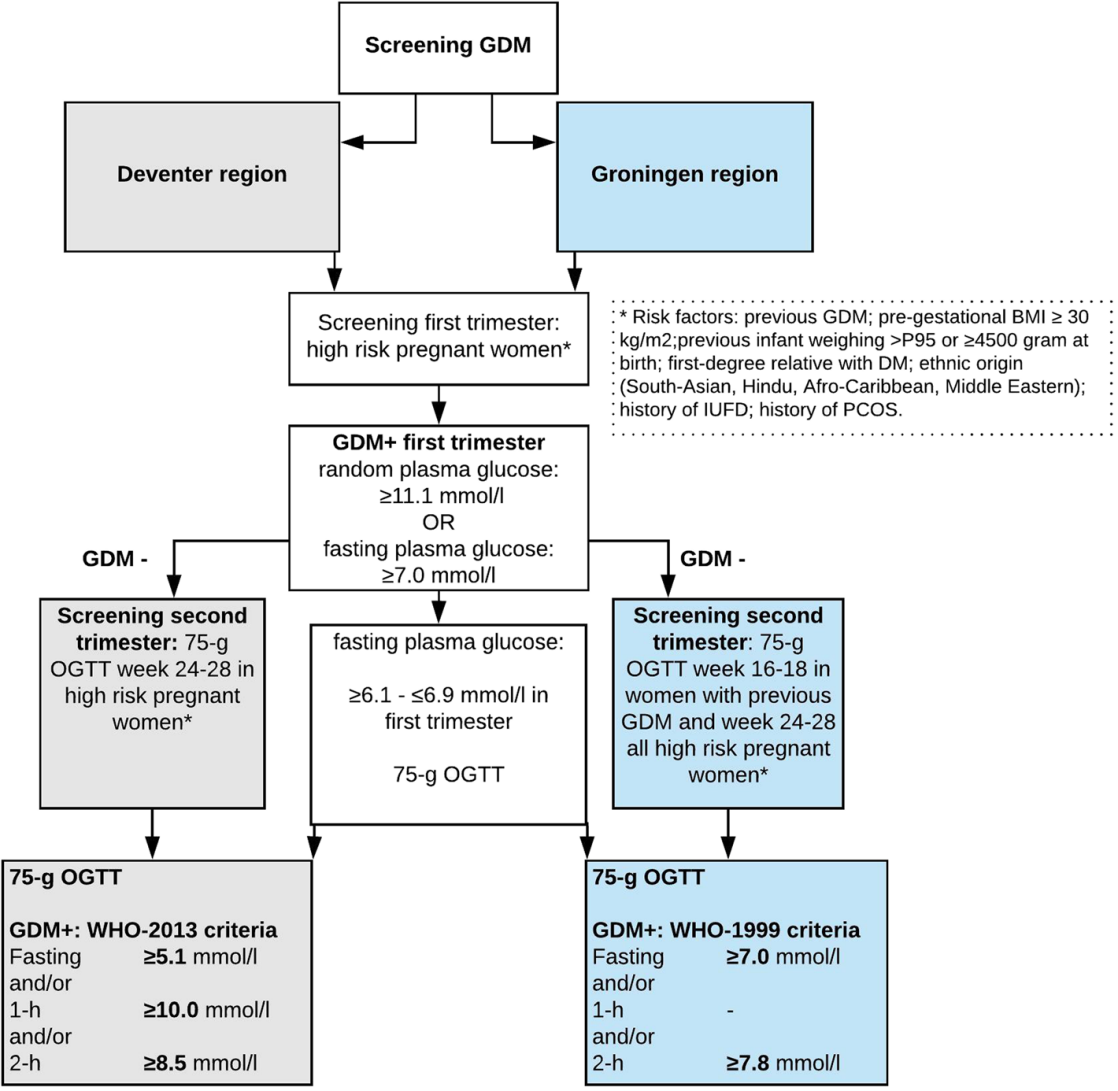
Screening and diagnosis of gestational diabetes. Abbreviations: GDM, gestational diabetes mellitus; WHO, World Health Organization; OGTT, oral glucose tolerance test; BMI, body mass index; DM; diabetes mellitus; IUFD, intra uterine foetal death; PCOS, polycystic ovary syndrome

Criteria for screening and diagnosis of GDM are summarized in Fig. 1 [16, 17]. After GDM diagnosis all women were treated based on the national guideline. First, all women received dietary counselling and instructions for self-monitoring of the blood glucose levels (SMBG). According to the guideline, insulin therapy was started if the blood glucose levels were repeatedly above the treatment targets (two blood glucose values above the treatment target at the same day) despite dietary treatment: fasting blood glucose level > 5.3 mmol/l and/or either a 1-h postprandial blood glucose level > 7.8 mmol/l, or 2-h postprandial blood glucose level > 6.7 mmol/l. Options for insulin therapy regimens were: ultra-short-acting insulin, once daily long-acting insulin, or a combination of both (basal-bolus). Metformin was occasionally prescribed in obese women (body mass index (BMI) > 30 kg/m^2^) in the Deventer hospital (depending on glycaemic control). Based on SMBG women were advised to adjust diet or increase insulin- or metformin dose to maintain blood glucose levels within the target range.

Women were seen at the obstetric outpatient clinic regularly and foetal growth was evaluated by ultrasonography at least every 4 weeks. Moreover, all patients were discussed every two to three weeks multidisciplinary. Based on similar guidelines in the two regions labour was induced between 38 and 39 weeks of gestation in women on insulin therapy or earlier on indication. In women with a diet, labour was induced between 38 and 40 weeks taking glycaemic control, estimated foetal weight and non-GDM related risk factors into consideration.

### Outcomes and definitions

All electronic medical- and birth records were retrospectively reviewed and data between 2011 and 2016 were included in an anonymised database. Maternal characteristics were age, ethnicity (Caucasian, Asian, African American, Mediterranean or unknown), parity, pre-gestational BMI, risk factors for GDM, hypertensive disorders, results of 75-g OGTT, and treatment details. Chronic hypertension was defined as a systolic blood pressure (SBP) ≥140 mmHg and/or a diastolic blood pressure (DBP) ≥90 mmHg at booking before 20 weeks of gestation, or the use of blood-pressure lowering drugs before pregnancy.

### Obstetric and neonatal outcomes

Obstetric outcomes collected were induction of labour, mode of delivery (spontaneous vaginal delivery, assisted vaginal delivery (vacuum extraction or forceps), intrapartum caesarean delivery or planned caesarean delivery), gestational age at birth, pregnancy-induced hypertension (PIH) and preeclampsia. PIH was defined as a SBP ≥140 mmHg and/or a DBP ≥90 mmHg, after 20 weeks of gestation in a previously normotensive woman. Preeclampsia was defined ad PIH plus the presence of proteinuria (≥300 mg/24-h) and also included women who had eclampsia and HELLP syndrome.

Neonatal outcomes were birth weight, large for gestational age (LGA; birth weight > 90th percentile, corrected for sex, ethnicity, parity, and gestational age) [21], small for gestational age (SGA; birth weight < 10th percentile, corrected for sex, ethnicity, parity, and gestational age) [21], preterm delivery (delivery before 37 weeks of gestation), 5 min Apgar score < 7, need for respiratory support, still birth/neonatal death, birth trauma (shoulder dystocia, fracture of humerus or clavicle, brachial plexus injury), neonatal hypoglycaemia, neonatal hyperbilirubinaemia, and admission to the neonatology department. Of note, neonates with extreme prematurity (delivery before 28 weeks of gestation, *n* = 3) were excluded prior to the analysis for the variable birth weight. Hyperbilirubinaemia was recorded if the neonate required treatment with phototherapy after birth. Neonatal hypoglycaemia (occurring > 2-h after birth) was defined as a blood glucose level < 2.6 mmol/l or treatment with glucose infusion [16]. Neonates born before 32 weeks (*n* = 2) of gestation with neonatal hypoglycaemia were excluded prior to the analysis as hypoglycaemia could well be caused by prematurity. Respiratory support was defined as the need for continuous positive airway pressure after birth or intubation.

### Statistical analyses

Statistical analyses were carried out using statistical package IBM SPSS (version 23.0. Armonk, NY: IBM Corp). Continuous variables are presented as mean ± standard deviation (SD) or as median and inter quartile range (IQR) according to the normal distribution status. Categorical variables are presented as numbers and frequencies (%). Appropriate (non)parametric tests were used to compare differences between the groups for continuous variables (independent *t*-test or Mann-Whitney *U*-test in case of skewed distribution) and categorical variables (Chi-square or Fisher’s exact test).

To examine the associations between the diagnostic groups and pregnancy outcomes, analyses were performed using logistic regression models in which the ORs and 95% CIs for the WHO-2013 group were calculated using the WHO-1999 group as reference group. Results were presented as unadjusted models and multivariable-adjusted models, with the multivariable-adjusted models adjusted for maternal age, pre-pregnancy BMI, ethnicity and parity. Only for the variables with sufficient statistical power multivariable-adjusted models were performed. The model analysing the association between the diagnostic groups and LGA-neonates was adjusted for maternal age and pre-pregnancy BMI. *P*-value < 0.05 was considered statistically significant.

## Results

Maternal characteristics are summarized in Table 1. A total of 1386 women with GDM were included in the study, 437 in the WHO-2013-cohort and 949 in the WHO-1999-cohort. In the WHO-2013-cohort, 49.4% of the women had GDM according to both the WHO-1999 criteria and WHO-2013 criteria. In the WHO-1999-cohort, 24.7% of the GDM women would not have had GDM according to the WHO-2013 criteria.

**Table 1:** Maternal characteristics of women diagnosed with gestational diabetes mellitus

	Cohort		
Characteristics	WHO-2013	WHO-1999	*P*-value*
N	437	949	
Age (years)	34.7 ± 5.1	32.1 ± 5.1	< 0.001
Ethnicity, n (%)			< 0.001
Caucasian	357 (81.7)	741 (78.1)	
Asian	9 (2.1)	72 (7.6)	
African-American	2 (0.5)	39 (4.1)	
Mediterranean	64 (14.6)	69 (7.3)	
Unknown	5 (1.1)	28 (3.0)	
Parity, n (%)			0.232
0	158 (36.2)	386 (40.7)	
1–2	242 (55.5)	499 (52.6)	
> 2	36 (8.3)	64 (6.7)	
First degree relative with DM, n (%)	82 (18.8)	376 (41.1)	< 0.001
History of PCOS, n (%)	10 (2.3)	50 (5.3)	0.011
History of GDM, n (%)	44 (10.1)	103 (10.9)	0.650
Previous infant weighing ≥4500 g at birth, n (%)	42 (9.6)	97 (10.2)	0.716
History of IUFD, n (%)	4 (0.9)	20 (2.1)	0.113
Pre-gestational BMI (kg/m^2^)	29.7 [26.0–34.4]	27.7 [24.2–31.8]	< 0.001
Pre-gestational BMI, n (%)			< 0.001
< 25 kg/m^2^	88 (20.8)	291 (31.5)	
25–29.9 kg/m^2^	129 (30.4)	288 (31.2)	
≥ 30 kg/m^2^	207 (48.8)	344 (37.3)	
Chronic hypertension, n (%)	8 (1.8)	43 (4.5)	0.013
Indication for OGTT, n (%)			< 0.001
Screening based on risk factors	362 (82.8)	650 (68.5)	
Diagnostic test based on symptoms/signs	66 (15.1)	270 (28.5)	
Unknown	9 (2.1)	29 (3.1)	
Diagnosis based on OGTT, n (%)^‡^	422 (96.6)	919 (96.8)	0.791
Gestational age at time of OGTT (weeks)	24.9 [23.3–29.0]	27.7 [25.9–30.7]	< 0.001
Gestational age at time of OGTT screening only (weeks)	24.4 [22.6–26.9]	27.3 [25.1–28.7]	< 0.001
Gestational age at time of OGTT diagnostic 3rd trimester only (weeks)	33.1 [28.7–35.3]	30.4 [27.7–33.6]	0.001
75-g OGTT			
Fasting glucose level (mmol/l)	5.3 [5.1–5.6]	5.0 [4.6–5.5]	< 0.001
1-h glucose level (mmol/l)	9.6 [8.0–10.5]	–	NA
2-h glucose level (mmol/l)	7.7 [6.6–9.0]	8.6 [8.1–9.4]	< 0.001
Diagnosis based on elevated fasting glucose level only, n (%)	170 (40.2)	8 (0.9)	< 0.001
Diagnosis based on elevated 2-h glucose level only, n (%)	46 (10.9)	877 (95.4)	< 0.001
Insulin treatment, n (%)	68 (15.6)	412 (43.4)	< 0.001
Metformin treatment, n (%)	14 (3.2)	–	NA

*Abbreviations*: *WHO* World health Organization, *BMI* body mass index, *DM* diabetes mellitus, *IUFD* intrauterine foetal death, *PCOS* polycystic ovary syndrome, *OGTT* oral glucose tolerance test, *NA* not applicable. Data are expressed as mean ± SD, median [IQR], or proportion of n (%)

*P-values were based on Student’s unpaired *t*-test (non-skewed continuous variables), Mann-Whitney U-Test (skewed continuous variables) or chi-square test (categorical variables)

^‡^Total number of women diagnosed with a 75-g OGTT. The other women (*n* = 45) were diagnosed with a random or fasting glucose level in first trimester of their pregnancy. Data with respect to first degree relative with DM 35 (3.7%) (WHO-1999-cohort), BMI 13 (3.0%) (WHO-2013-cohort) and 26 (2.7%) (WHO-1999-cohort), gestational age at time of OGTT 15 (1.6%) (WHO-1999-cohort), are missing

In total, 1341 women (96.4%) were diagnosed by OGTT and 45 (3.6%) women were already diagnosed in first trimester by a random or fasting glucose level. The median fasting glucose level was higher in the WHO-2013-cohort and the 2-h glucose level was lower, compared to the WHO-1999-cohort. GDM diagnosis was based on elevated fasting glucose level only in 40.2% in the WHO-2013-cohort, compared with 0.8% in the WHO-1999-cohort. GDM was diagnosed based on elevated 2-h value in 10.9% in the WHO-2013-cohort and in 95.4% in the WHO-1999-cohort. Women in the WHO-2013-cohort were diagnosed earlier in pregnancy (24.9 [IQR 23.3–29.0] vs. 27.7 [IQR 25.9–30.7] weeks) and less women had their OGTT performed based on symptoms or signs in third trimester (15.1% vs. 28.5%) instead of screening based on predefined GDM risk-factors. Of the 270 women in the WHO-1999-cohort diagnosed with GDM based on signs suggestive of GDM, 127 (47.0%) retrospectively appeared to have risk factors for GDM. Of these, 12 women tested negative on a first OGTT in the 2nd trimester and 115 women were not screened. In the WHO-2013-cohort 15.6% of the women received insulin treatment compared with 43.4% in the WHO-1999-cohort. In the WHO-2013-cohort, 14 (3.2%) women were treated with metformin.

### Obstetric and neonatal outcome

Table 2 summarizes the obstetric outcomes. In the WHO-2013-cohort there were more spontaneous deliveries (73.1% vs. 67.4%, adjusted OR 1.52 (1.15–2.01)), less planned caesarean deliveries (7.8% vs. 11.7%, OR 0.64 (0.43–0.96)). Median gestational age at birth was higher for women in the WHO-2013-cohort (39.0 vs. 38.3 weeks, *p* = < 0.001) and women in the WHO-2013-cohort were less like to have induced labour (59.3% vs. 63.9%, adjusted OR 0.76 (0.59–0.98). There were no differences between the groups with respect to assisted vaginal delivery and intrapartum caesarean delivery. Prevalence of PIH was higher in the WHO-2013-cohort, although no differences were seen between the two groups regarding incidence of preeclampsia.

**Table 2:** Obstetric outcomes of women diagnosed with gestational diabetes mellitus

	Cohort				
Outcome variable	WHO-2013	WHO-1999	P-value*	OR**	Adjusted OR**
N	437	949			
Induction of labour, n (%)	256 (59.3)	606 (63.9)	0.102	0.82 (0.65–1.04)	0.76 (0.59–0.98)
Delivery type, n (%)					
Spontaneous vaginal delivery	318 (73.1)	638 (67.4)	0.032	1.32 (1.02–1.70)	1.52 (1.15–2.01)
Assisted vaginal delivery	35 (8.0)	79 (8.3)	0.712	0.93 (0.61–1.40)	NA
Intrapartum caesarean delivery	48 (11.0)	121 (12.8)	0.365	0.85 (0.60–1.21)	NA
Planned caesarean delivery	34 (7.8)	111 (11.7)	0.029	0.64 (0.43–0.96)	NA
Gestational age at birth (weeks)	39.0 [38.3–39.6]	38.3 [38.0–39.0]	< 0.001	NA	NA
Pregnancy-induced hypertension, n (%)	50 (11.5)	61 (6.4)	0.001	1.89 (1.28–2.80)	1.71 (1.11–2.63)
Preeclampsia, n (%)	12 (2.8)	30 (3.2)	0.683	0.87 (0.44–1.71)	NA

*Abbreviations*: *WHO* World health Organization, *OR* odds ratios, *NA* not applicable. Data are expressed as mean ± SD, or proportion of n (%)

*P-values were based on Student’s unpaired *t*-test (non-skewed continuous variables), or chi-square test (categorical variables)

**OR, 95% confidence intervals were derived from logistic regression models using the WHO-1999 group as reference group. Multivariable adjustment included maternal age, pre-gestational body mass index, ethnicity and parity. When the statistical power of a variable was not sufficient or the outcome variable was continuous ‘NA’ was reported

Table 3 shows the neonatal outcomes. The percentage of LGA neonates (corrected for sex, ethnicity, parity, and gestational age) was lower in the WHO-2013-cohort (16.5% vs. 18.5%, adjusted OR 0.90 (0.66–1.25)), but this was not statistical significant. Birth weight was accordingly higher (3512 vs. 3399 g, *p* = < 0.001). Neonatal hypoglycaemia was more often diagnosed in offspring of the WHO-2013-cohort (9.6% vs. 4.2%, adjusted OR 2.48 (1.52–4.05))*.* There were no significant differences seen between the two groups with respect to neonatal hyperbilirubinaemia, preterm delivery, birth weight in categories, SGA, 5 min Apgar score < 7, need for respiratory support, birth trauma, still birth/neonatal death, and admission to the neonatology department.

**Table 3:** Neonatal outcomes of women diagnosed with gestational diabetes mellitus

	Cohort				
Outcome variable	WHO-2013	WHO-1999	*P*-value*	OR**	Adjusted OR**
N	437	949			
Preterm delivery, n (%)	27 (6.2)	60 (6.3)	0.934	0.98 (0.61–1.57)	NA
Birth weight (g)	3512 ± 459	3399 ± 532	< 0.001	NA	NA
Birth weight, n (%)			0.136	NA	NA
Infants < 4000 g	384 (87.9)	831 (87.8)			
Infants 4000–4499 g	42 (9.6)	104 (11.0)			
Infants ≥4500 g	11 (2.5)	11 (1.2)			
Large for gestational age, n (%) ^‡^	72 (16.5)	176 (18.5)	0.379	0.87 (0.65–1.18)	0.90 (0.66–1.25)
Small for gestational age, n (%) ^‡^	14 (3.2)	37 (3.9)	0.538	0.82 (0.44–1.54)	NA
5 min Apgar < 7, n (%)	7 (1.6)	32 (3.4)	0.068	0.47 (0.21–1.08)	NA
Respiratory support, n (%)	14 (3.2)	37 (3.9)	0.519	0.81 (0.44–1.52)	NA
Birth trauma, n (%)	15 (3.4)	30 (3.2)	0.791	1.09 (0.58–2.05)	NA
Hypoglycaemia, n (%)	42 (9.6)	40 (4.2)	< 0.001	2.41 (1.54–3.78)	2.48 (1.52–4.05)
Hyperbilirubinaemia, n (%)	4 (0.9)	24 (2.5)	0.062	0.36 (0.12–1.03)	NA
Still birth/neonatal death, n (%)	1 (0.2)	2 (0.2)	1.000	NA	NA
Admission to the neonatology department, n (%)	54 (12.4)	139 (14.6)	0.272	0.83 (0.59–1.16)	0.79 (0.55–1.14)

*Abbreviations*: *WHO* World health Organization, *OR* odds ratios, *NA* not applicable. Data are expressed as mean ± SD, or proportion of n (%)

^*^P-values were based on Student’s unpaired *t*-test (non-skewed continuous variables), or chi-square test (categorical variables)

^**^OR, 95% confidence intervals were derived from logistic regression models using the WHO-1999 group as reference group. Multivariable adjustment included maternal age, pre-gestational body mass index, ethnicity and parity. Large for gestational age was adjusted for maternal age and pre-gestational body mass index. Only for the variables with sufficient statistical power multivariable adjustment was performed. When the statistical power was not sufficient or the outcome variable was continuous ‘NA’ was reported

^‡^Corrected for sex, ethnicity, parity, and gestational age

## Discussion

This multicentre, retrospective cohort study shows the pregnancy outcomes in two cohorts applying different diagnostic criteria for GDM i.e. WHO-2013 and WHO-1999. Women in the WHO-2013-cohort had a higher pre-gestational BMI and more often PIH. However, they were diagnosed earlier, less often needed insulin therapy and had a higher percentage of spontaneous deliveries. No other differences in adverse obstetric and neonatal outcomes were seen between the two cohorts.

A number of previous international studies have addressed the effects of introduction of the WHO-2013 criteria on pregnancy outcomes [22–27]. They retrospectively studied pregnancy outcomes in women previously classified as non-GDM with other diagnostic criteria and newly defined as GDM with the WHO-2013 criteria [22–27]. These studies suggested that women newly diagnosed with the WHO-2013 criteria if untreated were at increased risk for adverse pregnancy outcomes, including PIH, preeclampsia, neonatal intensive care admission, caesarean section, shoulder dystocia, macrosomia and LGA neonates, compared to non-GDM women [22–27]. In contrast to the aforementioned studies, women in our study both diagnosed with WHO-2013 or WHO-1999 criteria were treated similarly according to our national guideline. Two comparable studies with regard to treatment and comparison of two diagnostic approaches (Carpenter-Coustan criteria compared with the WHO-2013 criteria) showed that the percentage of LGA neonates was lower in the WHO-2013-cohort [28, 29]. In addition, one study also showed a reduction in caesarean deliveries, PIH, and assisted delivery after implementation of the WHO-2013 criteria [29].

In terms of the likelihood of having an LGA neonate, we found no significant differences between women diagnosed having GDM on the WHO-2013 criteria and women diagnosed having GDM on the WHO-1999 criteria. However, the percentage LGA neonates was lower in the WHO-2013-cohort. The reduction of LGA neonates is an important treatment target in GDM, since LGA is associated with short- and long term complications for the neonate. There are several potential explanations for the lower rates of LGA neonates in the WHO-2013-cohort found in our study and others [28, 29]. Firstly, the WHO-2013 criteria included a new group of women: 40.2% of the women were only diagnosed based on the fasting glucose cut-off value compared to 0.8% in the WHO-1999-cohort. By applying the more strict WHO-2013 criteria the prevalence of GDM increases, including presumably more mild cases of GDM, resulting in a lower percentage of LGA neonates. Several other studies have demonstrated that implementation of the WHO-2013 increases the prevalence of GDM [4, 14]. Moreover, a lower percentage of women in our WHO-2013-cohort (15.6%) required additional insulin therapy compared with the WHO-1999-cohort (43.4%).

Secondly, women in the WHO-2013-cohort were screened and diagnosed with GDM earlier (WHO-2013-cohort: median ~ 25 weeks, WHO-1999-cohort: median ~ 28 weeks), so that group had earlier dietary or insulin intervention. More women in the WHO-1999-cohort were diagnosed based on signs suggestive of GDM (e.g. polyhydramnios/foetal macrosomia). Therefore the WHO-1999-cohort may include women with a more advanced stage of GDM leading to higher rates of LGA. Nevertheless, approximately 50% of all women diagnosed with GDM based on signs suggestive of GDM, retrospectively had a risk factor for GDM that justified 2nd trimester screening in the first place. However, even when we only considered women who were diagnosed based on 2nd trimester screening because of GDM risk factors, gestational age at diagnoses remained different between the groups. The earlier screening and diagnosis of GDM in the WHO-2013-cohort could have led to earlier treatment and therefore to a better outcome. Landon et al. also demonstrated that offering early treatment to women with modest degrees of hyperglycaemia in pregnancy results in reduction of foetal overgrowth [30].

The only obstetric parameters which differed between the two cohorts were the higher incidence of planned caesarean section and induction of labour in the WHO-1999-cohort. This may be due to difference in clinical obstetric practice between both regions. But may also be due to differences related to GDM including more estimated macrosomia on ultrasound, worse glycaemic control indicated by significantly more insulin therapy.

An increase in neonatal hypoglycaemia was seen in the WHO-2013-cohort. This can be explained by an active screening policy in all neonates in the hospital that used the WHO-2013 criteria unlike the “WHO-1999 hospitals”, that screened neonates by indication. This finding suggests that roughly 50% neonatal hypoglycaemia might be missed without active screening, potentially leading to long-term adverse outcomes. Moreover, in the WHO-2013-cohort a higher percentage of women were diagnosed with PIH. In the WHO-1999-cohort more women were diagnosed with chronic hypertension in first trimester of their pregnancy. This finding suggests that the difference in PIH between the WHO-2013-cohort and WHO-1999-cohort also can be explained by an earlier diagnosis of chronic hypertension in first trimester in the WHO-1999 cohort.

This study gives no information on differences in incidence of GDM between the two diagnostic approaches. In the WHO-2013-cohort, 50.6% of the women were positive for GDM according the WHO-2013 criteria only and 49.4% had GDM according to both the WHO-2013 criteria and WHO-1999 criteria. Both cohorts differed in some clinical characteristics: women in the WHO-2013 cohort were older, had a higher pre-gestational BMI and were more often diagnosed on the fasting glucose level compared with the WHO-1999-cohort. These factors are associated with a less favourable metabolic profile. Although the WHO-2013-cohort seemingly consisted of a group of women with milder glucose intolerance, they appeared to have a worse metabolic profile. It seems that the WHO-2013 criteria have a better ability to select women with a worse metabolic profile.

The main strength of this study is that it evaluates the pregnancy outcomes of women with GDM diagnosed by the old and new WHO-criteria in a real-life clinical setting. Moreover, after GDM diagnosis all women were treated equally based on the national guideline. Several potential limitations of this study should be noted. First, this study was conducted in three different hospitals in two regions of the Netherlands. It is possible that the study populations and obstetric management between the hospitals were different. One centre is a tertiary care centre and two are larger secondary care centres and this might have led to a selection bias. However, the only important difference between secondary care centres and tertiary care centres in the Netherlands is the referral function for deliveries under 32 weeks of gestational age for neonatal purposes. In all other aspects, population and care is comparable. Secondly, the study was limited by its retrospective study design and this resulted in missing data for some variables in the electronic medical- and birth records. Thirdly, the sample size was limited to find significant differences between the groups for relatively rare pregnancy outcomes, such as birth trauma, still birth/neonatal death, and preeclampsia. Due to the lack of statistical power for some pregnancy outcomes it was not possible to adjust these outcomes for possible confounding factors. Finally, this study gives no information on differences in incidence of GDM between the two diagnostic approaches since the exact number of pregnant women in the two populations is not known. The national guideline advocate targeted testing for GDM, and therefore we do not have data on universal testing.

## Conclusions

In summary, this study demonstrated that application of the WHO-2013 criteria was associated with a reduced need for insulin treatment and more spontaneous deliveries. Although an earlier diagnosis of GDM might contribute to these differences, milder GDM by selection is proposed to play a major role. No differences were found in adverse pregnancy outcomes between the two diagnostic approaches.

This study contributes to the current debate regarding the value of implementation of new WHO-2013 diagnostic criteria for GDM but cannot provide a definitive answer. The data of well conducted population-based randomised studies (and meta-analyses) directly comparing the two diagnostic approaches are necessary to determine whether treatment of women with mild GDM is beneficial and cost-effective. Moreover, there is more information needed whether women with a 2-h glucose value between ≥7.8 - ≤8.4 mmol/l can be safely left untreated.

## Abbreviations

BMI: Body mass index
DBP: Diastolic blood pressure
DM: Diabetes mellitus
GDM: Gestational diabetes mellitus
HAPO: Hyperglycemia and Adverse Pregnancy Outcomes
IADPSG: International Association of the Diabetes and Pregnancy Study Groups
IQR: Inter quartile range
LGA: Large for gestational age
OGTT: Oral glucose tolerance test
PIH: Pregnancy-induced hypertension
SBP: Systolic blood pressure
SD: Standard deviation
SGA: Small for gestational age
SMBG: Self-monitoring of the blood glucose levels
WHO: World Health Organization
